# Preferred habitat and effective population size drive landscape genetic patterns in an endangered species

**DOI:** 10.1098/rspb.2013.1756

**Published:** 2013-10-22

**Authors:** Byron V. Weckworth, Marco Musiani, Nicholas J. DeCesare, Allan D. McDevitt, Mark Hebblewhite, Stefano Mariani

**Affiliations:** 1 Faculties of Environmental Design and Veterinary Medicine, Universityof Calgary, Calgary, Alberta, CanadaT2N 1N4; 2 Wildlife Biology Program, Department of Ecosystem and Conservation Sciences College of Forestry and Conservation, University of Montana, Missoula, MT 59812, USA; 3 School of Biology and Environmental Science, University College Dublin, Belfield, Dublin 4, Republic of Ireland; 4 School of Environment and Life Sciences, University of Salford, Salford M5 4WT, UK; 5 College of Life Sciences, Peking University, Beijing 100871, People's Republic of China; 6 Montana Fish, Wildlife and Parks, 3201 Spurgin Road, Missoula, MT 59804, USA; 7 Panthera, New York, NY 10018, USA

**Keywords:** Canadian Rockies, genetic drift, habitat fragmentation, landscape genetics, least-cost paths, *Rangifer tarandus caribou*

## Abstract

Landscape genetics provides a framework for pinpointing environmental features that determine the important exchange of migrants among populations. These studies usually test the significance of environmental variables on gene flow, yet ignore one fundamental driver of genetic variation in small populations, effective population size, *N*_e_. We combined both approaches in evaluating genetic connectivity of a threatened ungulate, woodland caribou. We used least-cost paths to calculate matrices of resistance distance for landscape variables (preferred habitat, anthropogenic features and predation risk) and population-pairwise harmonic means of *N*_e_, and correlated them with genetic distances, *F*_ST_ and *D*_c_. Results showed that spatial configuration of preferred habitat and *N*_e_ were the two best predictors of genetic relationships. Additionally, controlling for the effect of *N*_e_ increased the strength of correlations of environmental variables with genetic distance, highlighting the significant underlying effect of *N*_e_ in modulating genetic drift and perceived spatial connectivity. We therefore have provided empirical support to emphasize preventing increased habitat loss and promoting population growth to ensure metapopulation viability.

## 1. Introduction

Maintaining wildlife habitat connectivity amidst the mosaic of human impacted landscapes has become a global conservation priority [1]. A lack of connectivity, particularly in the small isolated populations that typify many endangered species, leads to a multitude of demographic and genetic consequences. These include inbreeding depression [2], compromised immune response [3], loss of adaptive potential [4] and heightened susceptibility to demographic and environmental stochasticity [5]. Landscape genetics methodologies address the interactions between environmental features and the evolutionary processes such as gene flow, genetic drift and selection, and thus the mechanisms by which negative genetic impacts can be manifested. These methodologies are increasingly coupled with landscape resistance models to guide management decisions in identifying where best to set aside corridors, construct habitat linkages and otherwise promote connectivity [1,6,7].

Landscape resistance is a hypothesized measure of a landscape feature's impediment to gene flow. Studies using landscape resistance models often lack empirical data and instead rely heavily upon expert opinion to identify habitat variables important to resistance [6,7]. However, this qualitative approach has been shown to suffer from lack of repeatability and poor performance in describing actual landscape costs [8]. Some studies have begun to incorporate radio-telemetry data to identify key environmental metrics for constructing more objective landscape resistance surfaces [7,9]. A common method in studies of terrestrial animal ecology is to build models of resource selection functions (RSFs; e.g. [10]) to infer a species’ preferred habitat. Shafer *et al*. [9] have recently demonstrated the superiority of RSFs over null isolation-by-distance (IBD) and isolation-by-barrier models in predicting genetic structure. Prior limitations in landscape genetics research may be circumvented by using advances in empirically derived landscape resistance models.

While landscape genetics can enhance conservation planning, it cannot ignore the fundamental evolutionary processes that underlie metapopulation dynamics and their spatial and temporal scales. For example, few studies have taken into account the potential impact of population size on patterns of genetic diversity and the potential for genetic drift to obstruct resistance models from identifying important landscape genetic relationships, particularly when dealing with endangered species. Genetic drift is an evolutionary process with important implications in conservation biology due to its sensitivity to population fluctuations and temporal and geographical isolation [11–13]. The leading parameter that reflects evolutionary changes in population dynamics, such as drift, is the effective population size (*N*_e_), defined as the size of an idealized population exhibiting the same rate of random genetic drift as the population under consideration [14], and which can roughly be seen as the number of breeders that contribute genes across generations. The loss of genetic diversity caused by genetic drift is inversely proportional to *N*_e_, following approximately *H*_t_/*H*_0_ = [1–(1/2*N*_e_)]*^t^*, where *H*_t_/*H*_0_ is the reduction in heterozygosity after *t* generations. Consequently, knowledge of *N*_e_ can be a powerful tool in conservation as a predictor of genetic diversity loss, inbreeding and, perhaps most important to landscape genetics studies, population differentiation.

In this study, we conducted a detailed landscape genetics analysis that incorporates the effect of *N*_e_ among the factors determining patterns of genetic diversity. Specifically, we analyzed the primary environmental and demographic variables that drive ungulate population substructure in endangered woodland caribou (*Rangifer tarandus*). Our *a priori* landscape models were chosen to test specific hypotheses regarding the factors thought to have the greatest conservation impact on threatened species, including caribou. We test four hypotheses that population genetic structure is influenced by: (i) preferred habitat availability, (ii) anthropogenic barriers, (iii) predation risk, or (iv) reduced *N*_e_. If small populations are experiencing rapid genetic drift owing to small *N*_e_, this may explain a large variance component in population-pairwise genetic distances and obscure the signal from landscape variables. Woodland caribou offer an ideal species to explore these hypotheses as their ecological and conservation challenges are well documented in the literature [15–17] and genetic and spatial telemetry data are available for many populations [10,18].

## 2. Methods

### (a) Study area

**Figure 1.**
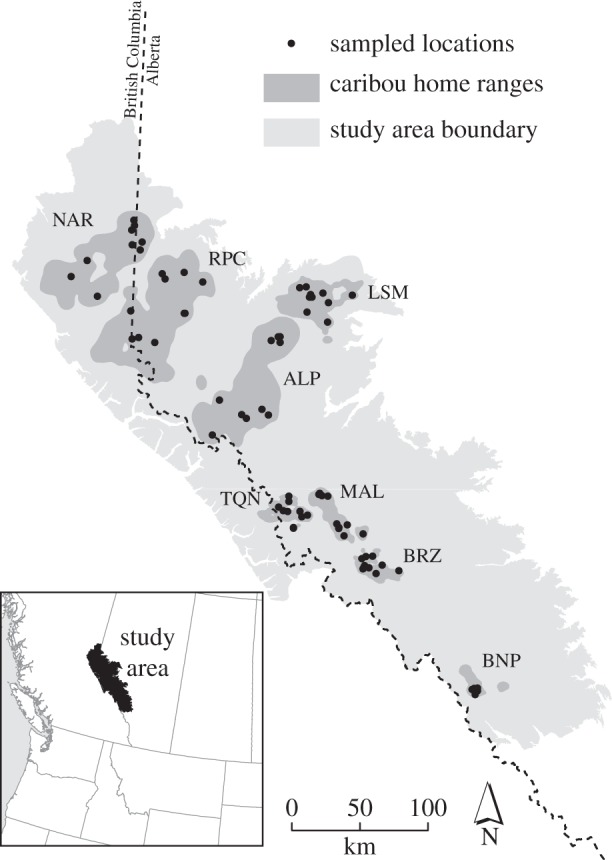
Map of study area in west-central Alberta. ‘Sampled locations’ for starting points of pairwise least-cost path simulations are represented by 10 points selected from caribou GPS locations per herd. Herd abbreviations follow table 1.

The study area encompassed an approximately 70 000 km^2^ region in west-central Alberta and eastern British Columbia, Canada (figure 1) that lies within the central Canadian Rockies ecosystem, and includes an approximately 16 000 km^2^ and 12 000 km^2^ of federally and provincially protected areas, respectively. The remaining area is primarily managed by provincial governments for natural resource extraction, including forestry, oil and natural gas industries. The topography is typified by the rugged slopes of the Rocky Mountains (400–3937 m) enveloping flat valley bottoms. The climate is characterized by long winters and short, dry summers and habitat types include montane, subalpine and alpine ecoregions that correspond to increasing elevation and decreasing annual productivity. Protected areas tend to be located in the more mountainous regions, in contrast to the areas of highest human impact occurring predominantly in the boreal foothill regions in the eastern portion of the study area. Roads, seismic lines, well pads and forestry cut blocks were more prevalent in the eastern portion of the study area.

We studied caribou herds representing eight spatially distinct populations (figure 1; A la Pêche, Banff, Brazeau, Little Smoky, Maligne, Narraway, Redrock Prairie Creek and Tonquin) that included both central mountain and boreal ecotypes. The study area represents the area historically available to these specific caribou herds [10]. Census herd size estimates (*N*_c_; table 1) were based upon population data from 2006 to 2009 [19].

**Table 1.** Population parameters for all caribou herds analysed; including name (Herd), herd abbreviation (abbr.), sample size (*n*), census population size (*N*_c_), effective population size (*N*_e_) with 95% confidence intervals (CIs), the ratio of *N*_e_ to *N*_c_ (*N*_e_/*N*_c_) and population inbreeding coefficient (*F*_IS_).

population	abbreviation	*n*	*N*_c_	*N*_e_ (CI)	*N*_e_/*N*_c_	*F*_IS_
Narraway	NAR	46	100	37.6 (32.3–44.3)	0.38	0.054
Redrock Prairie Creek	RPC	55	212	33.8 (29.2–39.5)	0.16	0.068
A La Peche	ALP	34	135	24.5 (20.9–29.1)	0.18	−0.008
Little Smoky	LSM	38	78	21.8 (18.6–26.0)	0.28	−0.015
Banff National Park	BNP	5	5^a^	n.a.	n.a.	−0.226
Brazeau	BRZ	6	10	4.4 (2.4–10.9)	0.44	−0.141
Maligne	MAL	5	4^b^	n.a.	n.a.	−0.020
Tonquin	TQN	18	74	35.5 (21.7–78.1)	0.48	0.012

^a^The Banff population is now extinct.

^b^The Maligne has declined further since sampling.

### (b) Genetic data

The 207 individuals analysed represent those herds previously analysed [18] for which validated habitat data were also available [10]. The genetic data used here are from individuals PCR-amplified and genotyped at 14 polymorphic microsatellite loci, following Weckworth *et al*. [18] (DRYAD entry doi:10.5061/dryad.gn22271h). DNA was derived from blood samples collected through agency monitoring efforts between 2001 and 2009.

### (c) Analysis of genetic data

For comparison with population-pairwise resistance distances (see below), we calculated standard pairwise estimates of *F*_ST_ [14] in Microsatellite Analyser v. 3.0 (MSA; [20]). We also used the MSA to calculate pairwise chord distance (*D*_c_, [21]). *D*_c_ emphasizes genetic drift over mutation, reflects decreases in populations better than other genetic distance metrics, and thus may be particularly suited for microsatellites and fine-scale landscape genetic analyses such as those used here [21,22]. Significance for *F*_ST_ was calculated using 10 000 randomizations, correcting for type I errors using sequential Bonferroni adjustment. We also estimated deviations of observed heterozygosities from those expected under Hardy–Weinberg equilibrium (HWE), using population inbreeding coefficients (*F*_IS_) using FSTAT 2.9.3 [23], where *F*_IS_ > 0 indicates greater inbreeding than expected under HWE and *F*_IS_ < 0 indicates greater heterozygosity than expected [2].

To calculate the *N*_e_ within each studied population, we estimated *N*_e_ using the linkage disequilibrium (LD) method in LDNe [24]. We report analysis results after excluding alleles with frequency less than 0.02 (as suggested by Waples & Do [12]) and with 95% confidence intervals derived from a jack-knife approach. Although our populations violate some of the assumptions of LDNe, such as having overlapping generations, Robinson & Moyer [25] demonstrate that the LDNe method performs well with relaxed assumptions. Additionally, *N*_e_ was used to provide a quantitative, non-landscape factor that may affect patterns of genetic differentiation among populations. We constructed a population-pairwise matrix of the harmonic mean of *N*_e_ between each population pair.

Finally, herds were assessed for genetic effects of rapid reduction in population size using the program BOTTLENECK [26]. A two-phase model of mutation (TPM) was assumed with multi-step mutations accounting for 5%, 10% and 20% of all mutations. We used the Wilcoxon signed rank test, which is suggested to provide the most robust statistical results for tests of bottlenecks for datasets with fewer than 20 loci [26].

### (d) Landscape resistance for caribou

We considered four landscape variables to be potentially biologically important in determining gene flow between caribou populations. These include a combination of ecological and anthropogenic factors that are explained below.

**Figure 2.**
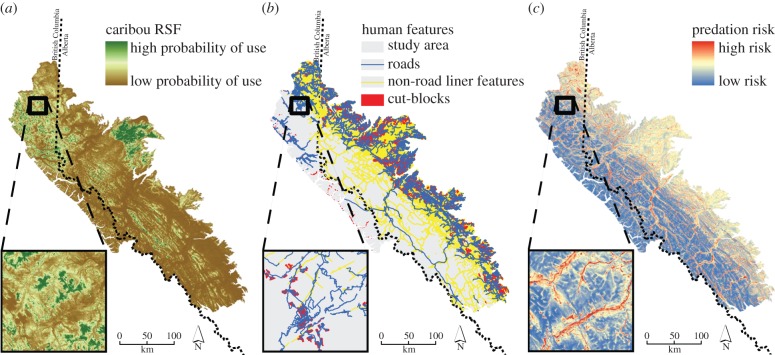
Maps depicting the baseline landscape variables used to calculate resistance surfaces. These include (*a*) the caribou RSF, (*b*) human features, which are here depicted together, but a separate resistance surface was calculated for each (roads, non-road linear features and cut blocks) and (*c*) predation risk from wolves. The inset of each map provides a 30 m pixel resolution of a subset of the baseline landscape variable.

Habitat loss and fragmentation has been cited as one key driver in caribou population declines [17]. We used a resource selection function (figure 2*a*, RSF; [10]) model that included multiple topographic (elevation, slope, aspect, topographic position, distance to water), climatic (percentage snow cover, distance to tree line) and vegetative (land cover type and normalized difference vegetation index) variables that are recognized as important predictors of caribou habitat suitability [15,16]. DeCesare *et al*. [10] estimated an RSF for the same caribou populations as considered here, including a ‘baseline’ RSF that excluded anthropogenic effects. For this analysis, we spatially applied the baseline RSF to our study area to estimate preferred habitat under ‘pristine’ conditions, that is, the resistance imposed by natural landscape heterogeneity alone (i.e. all contemporary human features removed; figure 2*a*).

Caribou use a strategy of spatial separation from wolves (*Canis lupus*) as a mechanism to avoid predation [27]. As such, we assessed the potential role of predation as a driver of landscape resistance using a previously developed spatial model of predation risk that integrated both the probabilities of encountering and being killed by wolves within our study area (figure 2*c*; [28]). Similar to our treatment of preferred habitat availability, we excluded the effects (coefficients) of anthropogenic features when applying the predation risk model to our study area to characterize predation-based resistance due to baseline natural conditions alone. Additionally, we treated three types of anthropogenic footprints (figure 2*b*), each as potential sources of landscape resistance, including: forestry cut blocks, roads and other non-road linear features (seismic lines and maintained hiking trails).

Finally, we evaluated the null hypothesis of a completely homogeneous landscape of resistance by relating population differentiation with geographical distance alone, in IBD analysis. It is important to note that while IBD is a common null model in landscape genetics studies that expresses the equilibrium between gene flow and genetic drift, it does not specifically account for the influence of genetic drift via demographic episodes of bottlenecking or founder events that are specifically related to population size.

**Figure 3.**
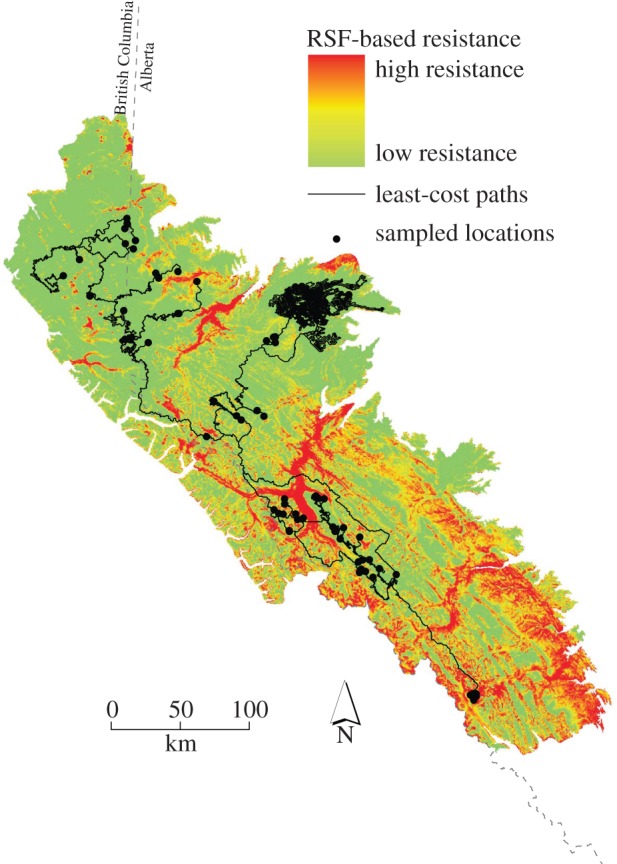
Map representing the optimized resistance surface for the caribou RSF that had the highest correlation with population-pairwise genetic distances. Black lines demonstrate examples of least-cost pathways from the PATHMATRIX simulations.

To determine whether landscape resistance has influenced past gene flow, and thus genetic differentiation among population pairs, we calculated cumulative cost distance of least-cost paths (LCP) between all pairwise population combinations for each hypothesized landscape resistance surface (figure 3). LCPs are modifications of geographical distances that reflect the hypothetical effects of landscape characteristics on promoting or impeding movement along a single pathway [29]. These movements represent dispersal, and so by comparing genetic divergence among individuals between cost distances, we can test hypotheses on the effects of landscape features and other environmental variables on gene flow [7]. The details of the LCP simulations and modelling are available in the electronic supplementary material.

### (e) Landscape genetic analyses

To evaluate caribou connectivity in this framework, we used simple and partial Mantel tests [30,31] to calculate the correlation between genetic distances and geographical distance, landscape resistance distances and harmonic means of *N*_e_. A review of the literature shows a recent emergence of diverse and computationally complex methodologies offered for landscape genetics analysis (reviewed in Balkenhol *et al*. [32]). However, the utility of these new analytical techniques remains unclear until further simulation studies can standardize their application. We used Mantel tests because they are easy to interpret, are widely used, retain a high level of power that is demonstrated to be appropriate for distance data [33,34] and are shown to correctly identify drivers of genetic diversity [35]. The statistical software package XLSTAT v. 2012.1.01 was used to perform all simple Mantel and partial Mantel tests to calculate Mantel's *r*. Significance of Pearson product–moment correlations was assessed using 10 000 permutations of the data. We used the Monte Carlo *p*-value to determine significant correlations.

We first tested for a pattern of IBD where genetic differentiation is predicted to increase with geographical distance as expected under mutation/migration/drift equilibrium [36]. We then tested for patterns of resistance for each of the landscape variables from the pairwise LCP analyses. Finally, we calculated partial Mantel's *r* between genetic distances and landscape resistance distances after accounting for the effects of geographical distance (IBD) and the effects of *N*_e_. This correlation provides a measurement of the strength of the environmental relationships after removing the influence of pure geographical distance and *N*_e_.

## 3. Results

### (a) Genetic analysis

*F*_ST_ and *D*_c_ population-pairwise genetic distances ranged from 0.021 to 0.213 and 0.227 to 0.603, respectively (see electronic supplementary material, table S1). As would be predicted, *F*_ST_ and *D*_c_ were highly correlated (*r* = 0.849, *p* < 0.0001). *F*_IS_ results (table 1) show a large negative value for BNP, which indicates an excess of heterozygosity. Most other populations trended towards heterozygosity expected under random mating.

Estimates of *N*_e_, were unclear in two herds (BNP and MAL) as 95% confidence intervals included infinity. These results are likely due to the small sample sizes of these herds (table 1). In order to obtain values for constructing the pairwise matrix of *N*_e_, we considered the results of the similarly sized population of BRZ, calculated the ratio of individuals estimated as effectively reproducing over the census population (*N*_e_/*N*_c_) and made the assumption that BNP and MAL had a similar ratio and from that calculated an *N*_e_ to use in the pairwise comparison matrix. For all other populations, the general trend showed that *N*_e_ was always lower than *N*_c_, and usually *N*_c_ was not included within the 95% *N*_e_ confidence intervals (table 1).

After correction for multiple comparisons (strict Bonferroni), significant excess heterozygosity (one-tailed Wilcoxon test for H excess) at TPM of 20% was detected in RPC and BNP herds. As the TPM converged towards a purely stepwise mutation model (TPM of 10% and 5%), only BNP continued to show significant heterozygosity excess (*p* < 0.003) expected under bottleneck scenarios.

### (b) Landscape analysis

**Figure 4.**
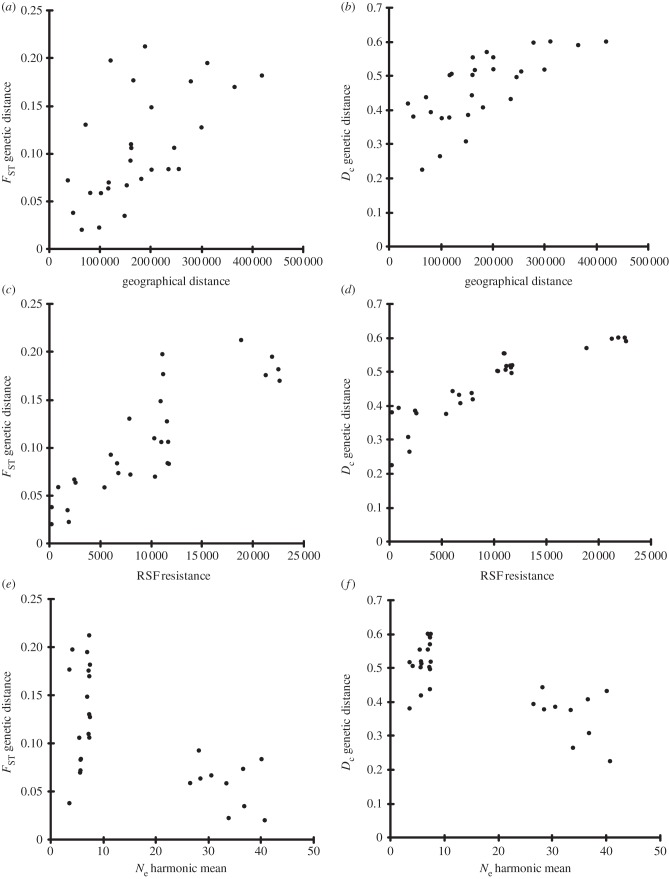
(*a,b*) Scatter plots from simple Mantel tests of geographical distance, (*c,d*) RSF resistance and (*e,f*) pairwise *N*_e_ harmonic mean for genetic distance metrics of *F*_ST_ and *D*_c_, respectively.

In the simple Mantel tests, all variables had positive significant correlations with both *F*_ST_ and *D*_c_ (table 2), except *N*_e,_ which was negatively correlated. Correlations were stronger (Mantel's *r*) in tests using *D*_c_ over *F*_ST_. Although there was a significant pattern of IBD (i.e. a correlation between genetic and geographical distances; figure 4), the strongest correlation for both *F*_ST_ and *D*_c_ was with the resistant distances based on the RSF (*r* = 0.856 and 0.900, respectively, and *p* < 0.0001 for both; table 2 and figure 4). *N*_e_ (table 2) had second highest correlations with genetic distances (*r* = −0.627, *p* < 0.0001, and −0.767, *p* < 0.0001, respectively; figure 4).

**Table 2.** Results of simple Mantel and partial Mantel tests for *F*_ST_ and *D*_c_. In partial Mantel tests, the variable controlled for in each test is given in parentheses. Statistical values reported include Mantel's *r* (*r*) and *p*-value (*p*). GEO, variables are geographical; RSF, resource selection function, representing preferred habitat; LIF, linear features; RDS, roads; CUB, cut blocks; PRR, predation risk areas and effective population size, *N*_e_.

	*F*_ST_		*D*_c_	
	*R*	*p*	*r*	*p*
simple Mantel				
GEO	0.599	0.0010	0.706	<0.0001
RSF	0.856	<0.0001	0.900	<0.0001
LIF	0.585	0.0020	0.704	<0.0001
RDS	0.623	0.0010	0.725	<0.0001
CUB	0.594	0.0010	0.705	<0.0001
PRR	0.595	0.0010	0.703	<0.0001
*N*_e_	−0.627	<0.0001	−0.767	<0.0001
partial Mantel (GEO)				
RSF	0.784	<0.0001	0.790	<0.0001
LIF	−0.045	0.8480	0.064	0.7640
RDS	0.225	0.2590	0.236	0.2260
CUB	−0.054	0.8270	0.021	0.9440
PRR	−0.119	0.5850	−0.084	0.6560
*N*_e_	−0.595	<0.0001	−0.834	<0.0001
partial Mantel (*N*_e_)				
GEO	0.563	0.0020	0.793	<0.0001
RSF	0.762	<0.0001	0.838	<0.0001
LIF	0.565	0.0020	0.819	<0.0001
RDS	0.554	0.0010	0.762	<0.0001
CUB	0.565	0.0020	0.804	<0.0001
PRR	0.563	0.0010	0.796	<0.0001
partial Mantel (RSF)				
GEO	−0.275	0.1530	−0.053	0.8010
LIF	−0.231	0.2380	0.031	0.8730
RDS	−0.141	0.4770	0.082	0.6700
CUB	−0.261	0.1800	−0.026	0.9050
PRR	−0.273	0.1600	−0.048	0.8190
*N*_e_	−0.222	0.2530	−0.596	0.0003

Partial Mantel tests that controlled for the effect of geographical distance (GEO) gave different results (table 2). Again, in all correlations, the Mantel *r*-values were greater in tests using *D*_c_ than *F*_ST_. After accounting for geographical distance, only the RSF-calculated distances and pairwise harmonic means of *N*_e_ remained significant. Although the correlation with RSF decreased slightly for both genetic metrics, the correlation with *N*_e_ increased, and surpassed the RSF, for *D*_c_.

In analyses that controlled for the effect of *N*_e_, partial Mantel *r*-values for all variables were significant. The *r*-values decreased for *F*_ST_ and increased for *D*_c_, except for RSF, which decreased for both genetic metrics (table 2). Nonetheless, the best correlation was consistently the RSF.

Finally, given the predominant relationship of genetic distance to the RSF distance matrix, we performed ad hoc partial Mantel tests on all variables, this time partialling out the variability explained by the RSF (table 2). In this case, the only variable significantly correlated was *N*_e_ with *D*_c_ (*r* = −0.596, *p* = 0.0003; table 2).

## 4. Discussion

Our study is one of the first to assess the impact of *N*_e_ in a landscape genetics framework. We found that after accounting for geographical distance, preferred habitat availability (figure 3, RSF) and *N*_e_ were the most significant explanatory variables in determining genetic distances between herds (table 2). This pattern was further supported when we controlled for RSF-based resistance and the partial Mantel *r*-values approached zero for most landscape variables (table 2), indicating, with one exception (*N*_e_), that no other variables explained genetic variability after accounting for preferred habitat availability. RSF models have only recently begun to be applied in landscape genetics, but are already demonstrating superior results to traditional landscape models [9].

After accounting for the effects of geographical distance, only the RSF and *N*_e_ were significant in explaining the variability among genetic relationships in both *F*_ST_ (RSF was best followed by *N*_e_) and *D*_c_ (*N*_e_ was best, followed by RSF; table 2). Similarly, in tests controlling for the variability explained by the RSF, only *N*_e_ (as measured by *D*_c_) was a significant explanatory variable. Interestingly, after controlling for *N*_e_, the genetic distance correlations with the anthropogenic variables increased for *D*_c_ (table 2). This improvement of correlation might simply be a statistical artifact owing to non-independence between some covariates (e.g. a suppressor effect [37]). However, this trend effectively reveals how, once the variance accounted for by *N*_e_ is partialled out, the residual variance is even more associated with all the gene-flow-related variables. Similarly, when the variance associated with RSF is removed, there remains a significant proportion explained by *N*_e_. Given the strong association between random drift and *N*_e_, our analysis starkly partitions genetic differentiation into a component caused by gene flow, and another, often overlooked, component that is due to random genetic drift; though predicted by theory [14], this pattern is very difficult to demonstrate in nature [1]. The interactive effects of drift and habitat fragmentation likely result in even stronger landscape fragmentation effects in small populations where drift is strongest; a detail that cannot be ignored in the precariously small populations that typify many endangered species [1].

The use of parameters related to population size have largely been absent from landscape genetics projects. However, ecological research on caribou found that censused population size was one of the key variables explaining genetic relationships [38,39], but did not explicitly test for landscape resistance at the same time. In Arctic char, Wollebæk *et al*. [40] found genetically assessed *N*_e_ to be the major cause of contemporary population differentiation. Thus, the effects of genetic drift due to small populations, rather than by geographical isolation due to distance, can be a major explanatory variable of population genetic relationships, and in some cases this may obfuscate the strength of relationships between genetic structure and landscape variables. This emphasizes that landscape genetics studies conducted without accounting for the effects of *N*_e_ are likely flawed and could result in misleading conservation recommendations.

The 50/500 rule often cited by conservation practitioners [41] postulates that a minimum of *N*_e_ = 50 is necessary to prevent a damaging rate of inbreeding in the short term, but that an *N*_e_ of 500–1000 is required for long-term genetic integrity [42]. Our figures of *N*_e_ were therefore concerning. Results from assignment tests [43] provide evidence of recent historical metapopulations in these caribou, a pattern that is also supported at the continental scale [18]. It is likely these same metapopulation dynamics allowed for the maintenance of genetic diversity through gene flow among demographically viable populations across unfragmented habitats. Given the persistently small populations sizes for these caribou, management strategies to protect them should emphasize fostering connectivity among caribou herds [18,43] and rebuilding past metapopulation relationships.

Despite the documented decline in population sizes for all caribou populations [19], tests for excess heterozygosity, as an indicator of recent bottlenecks, was detected consistently only for the Banff (BNP) population, and is corroborated by a negative *F*_IS_ value that is also indicative of heterozygosity excess (table 1; [2]). The Brazeau (BRZ) and Maligne (MAL) populations were of similar size, but there is some evidence that these two herds have recently exchanged migrants, which would effectively buffer them from the potential impacts of a population reduction [13], and explain non-significant heterozygosity excess. The Banff population suffered local extinction from an avalanche in 2009 [5]. Barring an increase in population size, both BRZ and MAL are at immediate risk of similar stochastic extinction [44].

Predation by wolves has been noted as the most important proximate threat to the persistence of threatened caribou populations [45]. Caribou natural history characteristics describe anti-predation behaviour through geographical spatial separation [27,46]. As such, a historic pattern of avoiding specific regions that provide good habitat for wolves could provide a natural barrier to gene flow. However, in our study, the correlation of predation risk to genetic distances was highly autocorrelated with geographical distance (table 2). Thus, predation by wolves may be too ephemeral to become a permanent landscape barrier that would influence gene flow. Alternatively, our model of predation risk may reflect wolf occurrence on a human manipulated landscape that is too recent to show up in genetic signatures. Anthropogenic barriers are frequently cited as major concerns for connectivity of fragmented populations [1,6]. With caribou, human-mediated landscape changes are predicted to be a major influence on population structure [45], particularly in Alberta [19]. However, similar to wolf predation in our study, after accounting for geographical distance or RSF, the relationship between anthropogenic features and genetic distance mostly disappeared (partial Mantel tests, table 2). The lack of a strong relationship independent of geographical distance may be due to the time lag of a genetic response to the anthropogenic features [47,48]. In caribou, a detectable numerical response to human land use changes have been documented to take several decades [49] and the potential negative impacts of anthropogenic features, even at the low density revealed here, cannot be dismissed.

The implications for endangered species such as woodland caribou are twofold: (i) in the threatened populations analysed here, modern anthropogenic features do not appear to yet have significant impacts on gene flow by themselves, but have been shown to reduce population size [38], thus leading to increased drift; (ii) conservation efforts should focus on preserving preferred caribou habitat to maintain the natural pattern of landscape resistance in caribou metapopulation dynamics.

## 5. Conclusion

Promoting connectivity among populations of threatened species in heterogeneous landscapes impacted by human disturbance is further complicated by the fact that most species already exist within discontinuous mosaics of preferred habitat [1,9,38]. Here, we demonstrated that the greatest predictor of genetic connectivity in caribou of west-central Alberta is preferred habitat availability. The distribution of preferred habitat demonstrated in figure 2*a*, and the associated resistance surface of that habitat (figure 3) exhibit the classic matrix of suitable habitat interwoven within a matrix of unsuitable space on which we would expect metapopulation dynamics to operate [50].

Metapopulation theory dictates that throughout the metapopulation, localized extinctions take place at the population level, only to be recolonized in the future. A reduction in connectivity (e.g. by habitat destruction or landscape barriers) lowers per patch immigration rate, thus inhibiting the rescue effect [50] and resulting in declines in abundance and occupancy of remaining patches [51,52]. For caribou, telemetry data (for females) indicated little movement between populations [38,43], suggesting a breakdown in such metapopulation dynamics. The Banff population illustrates the danger of decoupling metapopulation dynamics, as exemplified by its persistent isolation for decades with no new migrants, which ultimately resulted in stochastic localized extinction [5] and loss of that patch's genetic contribution to the metapopulation. Our results therefore emphasize the importance of habitat within and between population ranges for the viability of the metapopulation and its discrete elements.

The correlation that we found between genetic differentiation and low population numbers provides an empirical link between habitat loss and fragmentation [1,6]. The failure of demographic rescue in local populations points to lack of preferred habitat between populations and to a paucity of effective migrants, which, in turn, may be due to a synergistic relationship between declining caribou numbers and population density-dependent dispersal behaviour [38]. Little is known about the dispersal patterns of male caribou, but the lack of female dispersal in caribou is directly correlated with small isolated subpopulations throughout the Canadian Rockies [38,39,43]. In our study, after preferred habitat, the second variable that best explained levels of genetic structure was *N*_e_ (table 2), and if drift continues in these declining and isolated populations, barring any demographic rescue, we would predict that *N*_e_ will tend to explain an increasingly larger proportion of the variability.

Population size, and in particular *N*_e_, is an important variable that tends to be neglected in most landscape genetic studies [53]. Genetic drift due to small, isolated, populations can lead to spatial structuring in markers such as microsatellites that are frequently used in landscape genetic research. A failure to specifically account for the *N*_e_ component of genetic drift may lead to erroneous inferences of population structure strictly based on landscape features and, as a result, will fail to pinpoint crucial demographic processes important to conservation.
